# Brain-gut axis and psychiatric disorders: A perspective from bibliometric and visual analysis

**DOI:** 10.3389/fimmu.2022.1047007

**Published:** 2022-11-16

**Authors:** Pan Chen, Ling Zhang, Yuan Feng, Yu-Fei Liu, Tong Leong Si, Zhaohui Su, Teris Cheung, Gabor S. Ungvari, Chee H. Ng, Yu-Tao Xiang

**Affiliations:** ^1^ Unit of Psychiatry, Department of Public Health and Medicinal Administration, & Institute of Translational Medicine, Faculty of Health Sciences, University of Macau, Macao, Macau SAR, China; ^2^ Centre for Cognitive and Brain Sciences, University of Macau, Macao, Macau SAR, China; ^3^ The National Clinical Research Center for Mental Disorders & Beijing Key Laboratory of Mental Disorders, Beijing Anding Hospital & the Advanced Innovation Center for Human Brain Protection, Capital Medical University, Beijing, China; ^4^ School of Public Health, Southeast University, Nanjing, China; ^5^ Center on Smart and Connected Health Technologies, Mays Cancer Center, School of Nursing, UT Health San Antonio, San Antonio, TX, United States; ^6^ School of Nursing, Hong Kong Polytechnic University, Hong Kong, Hong Kong SAR, China; ^7^ University of Notre Dame Australia, Fremantle, WA, Australia; ^8^ Division of Psychiatry, School of Medicine, University of Western Australia / Graylands Hospital, Perth, WA, Australia; ^9^ Department of Psychiatry, The Melbourne Clinic and St Vincent's Hospital, University of Melbourne, Richmond, VIC, Australia

**Keywords:** psychiatric disorders, bibliometric analysis, gut microbiota, hotspots, brain-gut axis

## Abstract

**Background:**

The Brain-Gut Axis, a bidirectional signaling pathway that connects the intestinal and central nervous systems, plays an important role in the development of psychiatric disorders. However, the overall research trends in this field are unclear. This study explored the patterns of research on the brain-gut axis and psychiatric disorders from a bibliometric perspective.

**Methods:**

Relevant data were retrieved from the Web of Science Core Collection, with search terms on psychiatric disorders and the brain-gut axis. R (version 4.2.0), VOSviewer (version 1.6.17), CiteSpace software, and the online bibliometric platform were used in the data analysis.

**Results:**

A total of 2,298 articles published between 1993 and 2022 were identified, showing an increasing trend over time. China (1,859; 20.70%) was the country that contributed the most publications. The journal Nutrients (95; 4.13%) published the most publications. Cryan JF (153; H-index=73) and University College Cork (559; 22.54%) were the most influential author and the most productive institution, respectively. The high-frequency keywords were clustered into six themes, including neurodegenerative diseases, stress-related diseases, immune, brain behavior, depression, and probiotic-related topics; of which, depression (880; 2019), anxiety (207; 2018) and autism (191; 2019) were the most studied psychiatric disorders in the past 5 years. “Depressive symptom” (2019-2020) and “probiotic treatment” (2019-2020) were the main areas addressed in recent years.

**Conclusion:**

Research on the brain-gut axis and psychiatric disorders has attracted increasing attention in the past decade, with most publications originating from high-income level countries. This study provides a useful perspective on understanding the research trends, key hot topics, and research gaps in this expanding field.

## 1 Introduction

Brain-gut axis is a bidirectional signaling pathway that connects the intestinal and central nervous systems (CNS) (1). The links along this pathway also involve the autonomic nervous system (ANS), hypothalamic-pituitary-adrenal (HPA) axis, and enteric nervous system (ENS), and play an important role in maintaining homeostasis and regulating the normal function of the human body that involves neural, endocrine and immune systems (2). The brain can modulate gastric secretion, gastrointestinal (GI) motility (3) and sensory function of the gastrointestinal system (GI) (4). Conversely, evidence indicates that the GI system can affect brain functions involving cognition, behavior, nociception and emotional states (5, 6). Further, the highly diverse and varied gut microbiota appears to be the key modulator in the bidirectional interactions between the gut and brain (6, 7), which is not only locally connected with intestinal cells and ENS but is also directly associated with CNS *via* neuroendocrine and metabolic pathways (6). A number of studies have found that dysfunction of the brain-gut axis is associated with a higher risk of GI-related neuropsychiatric and neurological problems such as inflammatory bowel disease (IBD), irritable bowel syndrome (IBS), migraine headache (8), epilepsy (9) and Parkinson’s disease (7).

Psychiatric disorders often involve substantial disturbances in cognition, emotional regulation, or behavior, and are associated with a huge disease burden (10–13). Among these disorders, anxiety and depression are among the most common (10, 14, 15), affecting 301 million and 280 million people worldwide, respectively (16). Previous studies have found that the global burden of psychiatric disorders accounts for 32.4% of years lived with disability and 13.0% of disability-adjusted life-years (17). The etiology of psychiatric disorders is complex and associated with genetic, environmental, individual, family and social factors (16).

In recent years, as the association between microbiological factors and psychiatric disorders is better understood, the brain-gut axis is now viewed as an important mechanism in regulating mental health (18–20). Previous studies have explored the role of the brain-gut axis in various psychiatric disorders such as anxiety, depression (21), autism spectrum disorders (ASD) (22) and schizophrenia (9). For instance, depression and anxiety disorders are associated with the dysfunction of the HPA axis (23) which is the core endocrine stress system that regulates hormone secretion such as cortisol (24, 25). Animal model studies have found that stress disturbance could reshape the composition of gut microbiota, which could in turn regulate the HPA axis (26) and pivotal neurotransmitter systems (27). The hypothesis of “leaky gut” might explain the inflammation response in psychiatric disorders associated with increased gastrointestinal permeability (9). Previous studies also found that emotional states were related to GI motor function (28). For example, most children with ASD present with gastrointestinal symptoms that are likely caused by abnormalities in carbohydrate digestion and absorption, increased intestinal permeability, and marked differences in microbiota composition compared with non-ASD individuals (22, 29). In addition, some studies on the interaction between the brain-gut axis and schizophrenia have found that individuals with schizophrenia have an impaired intestinal barrier, increased bacterial translocation, and frequent gastrointestinal comorbidities (9). Therefore, it can be concluded that the brain-gut-axis plays an important role in various psychiatric disorders, and as such provides new possibilities for on the treatment of psychiatric disorders.

Bibliometric analysis is a commonly used method that presents the intellectual structure and emerging trends of a research topic or field by summarizing substantial bibliometric data quantitatively and qualitatively (30). Unlike traditional review methods, it can be used when the scope of the research area is broad. Further, it combines performance analyses, that examine the contributions of various research constituents (e.g., year, country, institution, author), and science mapping, that examines the relationships between each research constituent (30).

To date, despite the abundance of research on the brain-gut axis and psychiatric disorders (1, 18, 21, 22), the overall research trends of the brain-gut axis and psychiatric disorders are unclear. To fill this gap, this study comprehensively explored the patterns of research on the brain-gut axis and psychiatric disorders from a bibliometric perspective.

## 2 Methods

### 2.1 Data source and search strategy

**Figure 1:**
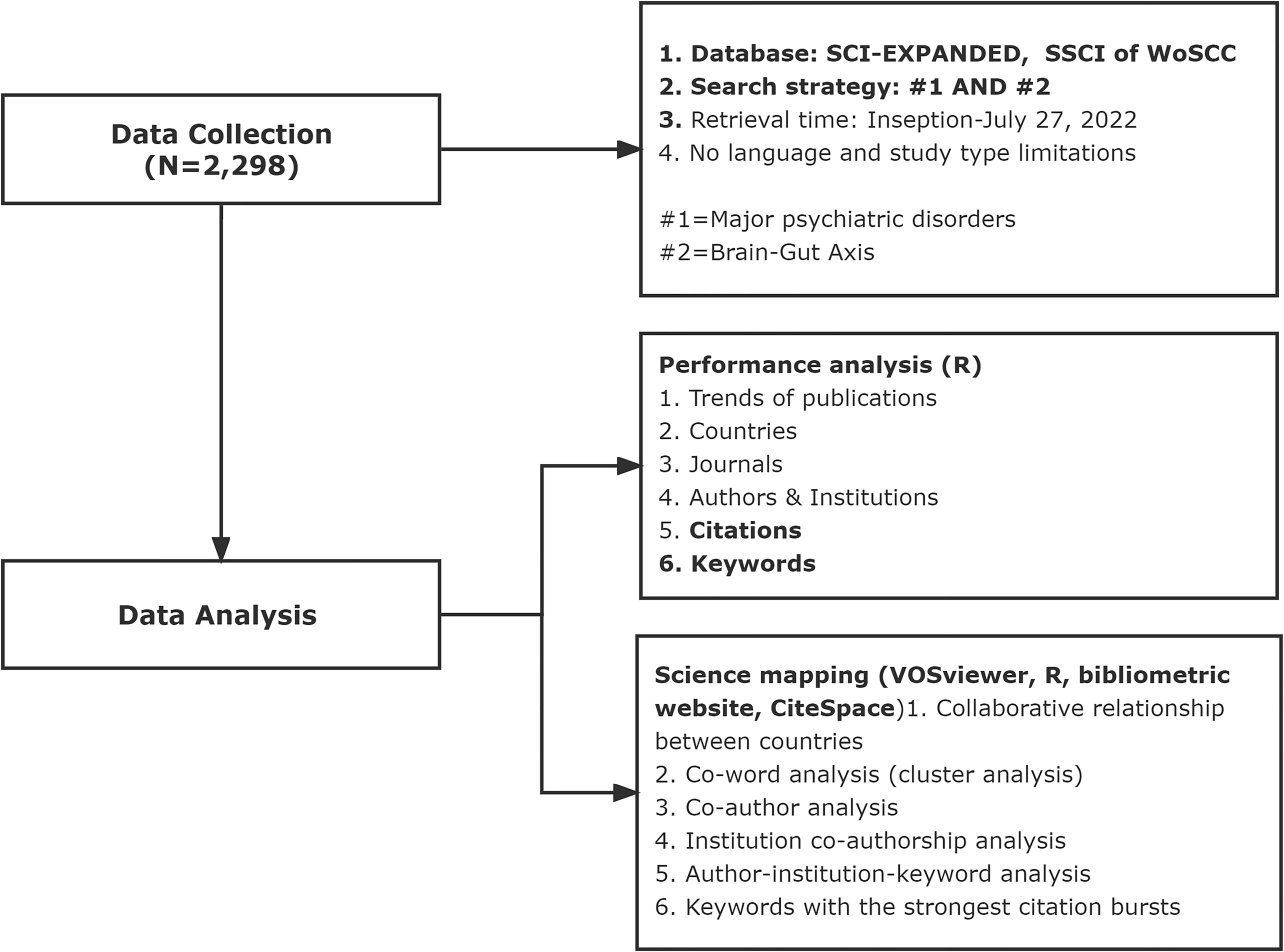
Flowchart of data collection and study design.

Relevant data were retrieved from the Science Citation Index Expanded (SCI-EXPANDED) and Social Sciences Citation Index (SSCI) in the Web of Science Core Collection (WoSCC) (30, 31) from its inception to July 27, 2022, with search terms on psychiatric disorders and Brain-gut axis as recommended previously (32). The search strategy was as follows: “mental* OR psychiatr* OR neuropsych* OR “depressi*” OR MDD OR anxi* OR “bipolar disorder*” OR mania OR manic OR “mood disorder*” OR “affective disorder*” OR “feeding and eating disorder” OR anorexia OR “eating disorder” OR “neurocognitive disorders” OR “neurodevelopmental disorder*” OR “personality disorder*” OR schizophren* OR schizoaffect* OR psychotic OR psychosis OR “sleep wake disorders” OR “substance abuse” OR “substance dependence” OR “alcohol use” OR “trauma and stressor related disorders” OR post-traumatic* OR PTSD OR autis* OR “attention deficit” OR ADHD OR “obsessive compulsive” OR OCD”. The data were extracted from included publications, exported as the format of “Plain text file” or “Tab-delimited file”, and then recorded as “Full record and cited references”. No limitations on language and publication type were applied. The flow chart of data collection is shown in **Figure 1**.

### 2.2 Data analysis

The method of synthetic analysis (33) was adopted to examine the trends of research on the brain-gut axis and psychiatric disorders both quantitatively and qualitatively. R program (version 4.2.0), VOSviewer (version 1.6.17) and CiteSpace Softwares (6.1.R2) (34) and the online bibliometric platform were used in the data analysis.

*Bibliometrix* package in R software (35) was used to analyze the patterns and characteristics of publications (e.g., number/citation, source, and authorship). Additionally, the relationship between three different fields (authors, institutions and keywords) was visualized using the unique Sankey diagram (36). The h-index and 2021 impact factor (IF) were used to evaluate the quality of publications and journals, respectively (37). The *mgcv* package in R software was applied to predict the future trend of publications in brain-gut axis and psychiatric disorders in the coming decade based on a generalized additive model (33, 38). The collaborative relationship between countries was visualized by the *Bibliometric* website (https://bibliometric.com/).

*VOSviewer* software is widely used for science mapping (39), which visualizes the collaborative relationships between authors, institutions, and the research topics in the field of Brain-Gut Axis and psychiatric disorders. A node in maps represents an author, an institution, or a keyword, and the size reflects the number of publications (NP) published by an author or an institution, or the occurrences of keywords. The thickness of edges linked by nodes represents the strength of the associations between nodes, which can also be described using the total link strength (TLS) (40). Different colors reflect various clusters. Furthermore, colors are ranked from blue to yellow by the average publication year (APY) of articles in the overlay maps. The keywords burst detection generated by CiteSpace software (41) was used to examine the hotspot’s evolution over time and predict the future frontiers in a certain field.

## 3 Results

### 3.1 Distribution of annual publications and citations

**Figure 2:**
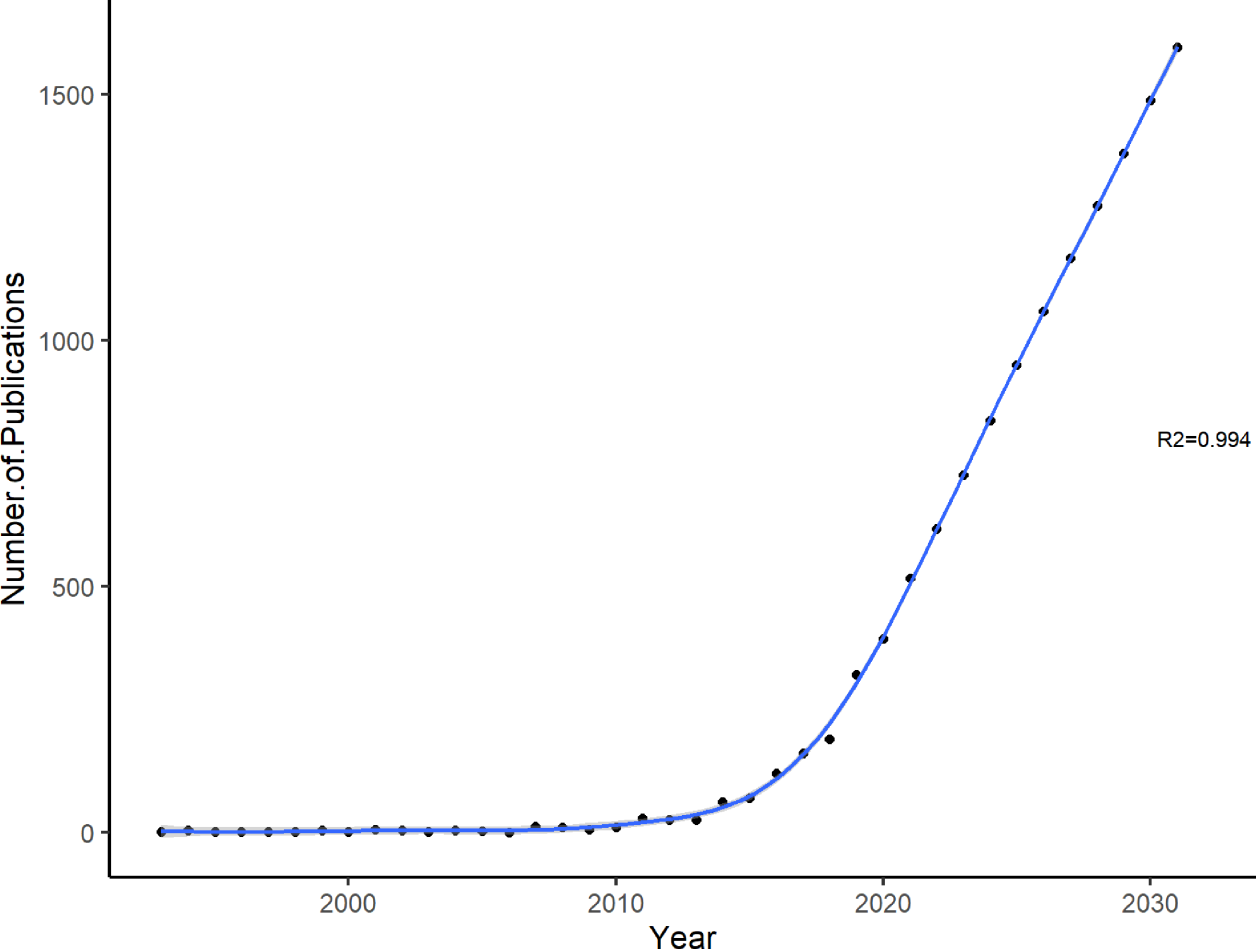
Trends of publications on brain-gut axis and psychiatric disorders.

A total of 2,298 articles published between 1993 and 2022 were identified. **Figure 2** shows a rapidly growing trend of publications on the brain-gut axis and psychiatric disorders, with a sharp increase since 2013. **Table S1** presents in detail the numbers and citations of relevant publications over time. The number of publications on the brain-gut axis and psychiatric disorders is predicted to increase to 1,059 by 2026 and to 1,704 by 2032.

### 3.2 Countries

**Figure 3:**
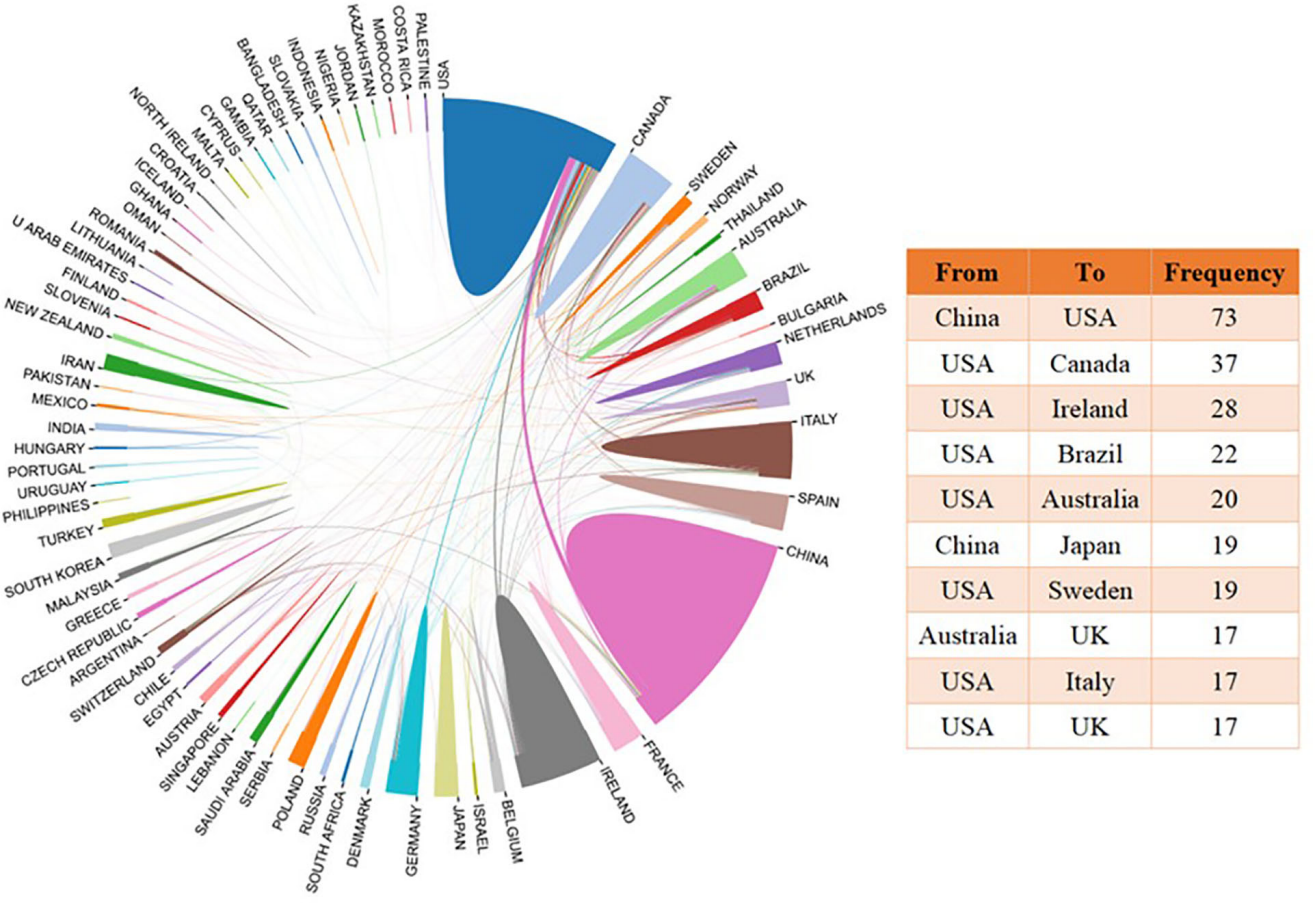
Collaborative research between countries on brain-gut axis and psychiatric disorders.

There were 69 countries involved in publications on the brain-gut axis and psychiatric disorders. **Figure S1** shows the distribution of publications by country. China (1,859; 20.7%), the USA (1,790; 19.93%), Ireland (653; 7.27%), Italy (510; 5.68%), Canada (452; 5.03%), Spain (320; 3.56%), Germany (293; 3.26%), Australia (291; 3.24%), France (283; 3.15%) and Japan (212; 2.36%) were the top 10 countries with the most publications. Nine of them were high-income countries according to the World Bank’s criteria (42). **Figure 3** shows the collaborative network between countries. The collaborations were most common between China and the USA (73; 3.18%), followed by between the USA and Canada (37; 1.61%), and between USA and Ireland (28; 1.22%).

If only corresponding authors were counted, 60 countries were involved in publications on the brain-gut axis and psychiatric disorders. **Table S2** shows the distribution of the top 10 countries of corresponding authors. Similarly, China (458; 19.93%), the USA (436; 18.97%), and Ireland (153; 6.66%) were ranked as the top three most productive countries. Publications from Ireland had the most citations (21,334). A higher ratio of multiple country publications (MCP) showed stronger multinational collaborations between countries (43). Among the top 10 countries, Australia had the strongest collaborations with other countries (MCP ratio=0.448), while the USA had the strongest internal collaborations (MCP ratio=0.188) (**Figure S2**).

### 3.3 Journals

The 2,298 identified publications were published in 687 journals. **Table 1** shows the top 10 journals with the most publications. The journal Nutrients (95; 4.13%; IF=6.706) published the most publications on the brain-gut axis and psychiatric disorders, followed by Brain Behavior and Immunity (69; 3.00%; IF=19.227), and Frontiers in Psychiatry (49; 2.13%; IF=5.435). The 2021 impact factors (IFs) of the top 10 journals ranged from 3.96 to 19.227. Based on the Journal Citation Reports (JCR), 40% of the 10 journals were classified as Q1 and others as Q2.

**Table 1:** Top 10 most productive journals on brain-gut axis and psychiatric disorders.

Sources	NP	Percent	IF (2021)	Category (JCR)
Nutrients	95	4.13%	6.706	Nutrition & Dietetics (Q1)
Brain Behavior and Immunity	69	3.00%	19.227	Immunology (Q1); Neurosciences (Q1); Psychiatry (Q1)
Frontiers in Psychiatry	49	2.13%	5.435	Psychiatry (Q2)
Frontiers in Neuroscience	41	1.78%	5.152	Neurosciences (Q2)
International Journal of Molecular Sciences	40	1.74%	6.208	Biochemistry & Molecular Biology (Q1); Chemistry, Multidisciplinary (Q2)
Scientific Reports	39	1.70%	4.996	Multidisciplinary Sciences (Q2)
Neurogastroenterology and Motility	35	1.52%	3.96	Clinical Neurology (Q2); Gastroenterology & Hepatology (Q3); Neurosciences (Q2)
Psychoneuroendocrinology	27	1.17%	4.693	Endocrinology & Metabolism (Q2); Neurosciences (Q2) Psychiatry (Q2)
Progress in Neuro-Psychopharmacology & Biological Psychiatry	26	1.13%	5.201	Clinical Neurology (Q2); Neurosciences (Q2); Pharmacology (Q2); Psychiatry (Q2)
Frontiers in Cellular and Infection Microbiology	24	1.04%	6.073	Immunology (Q2); Microbiology (Q1)

NP, Number of publications.

### 3.4 Authors and institutions

A total of 9,788 authors from 2,480 institutions were involved in the publications on the brain-gut axis and psychiatric disorders. **Table 2** shows the top 10 most active authors and institutions. The top three most influential authors were from University College Cork, namely Cryan JF (153; 6.66%; H-index=73) with a total of 24,814 citations, followed by Dinan TG (132; 5.74%; H-index=68) and Clarke G (57; 2.48%; H-index=39). University College Cork (559; 22.54%) was the most productive institution, followed by Chongqing Medical University (152; 6.13%) and McMaster University (134; 5.40%).

**Table 2:** Top 10 most active authors contributing to research on brain-gut axis and psychiatric disorders.

SCR	Author (N = 9,788)	H-index	TC	NP	Institution (N = 2,480)	NP	Percent
1	Cryan JF (University College Cork)	73	24,814	153	University College Cork	559	22.54%
2	Dinan TG (University College Cork)	68	22,937	132	Chongqing Medical University	152	6.13%
3	Clarke G (University College Cork)	39	10,861	57	Mcmaster University	134	5.40%
4	Stanton C (University College Cork)	26	4,695	35	University of California Los Angeles	89	3.59%
5	Stengel A (Charite Universitatsmedizin Berlin)	14	479	25	University of Melbourne	70	2.82%
6	Mayer EA (University Of California Los Angeles)	13	2613	22	University of California, San Diego	63	2.54%
7	Xie P (Chongqing Medical University)	13	1854	22	Zhejiang University	56	2.26%
8	Hashimoto K (Chiba University)	15	770	21	University of North Carolina Chapel Hill	54	2.18%
9	O'Mahony SM (University College Cork)	19	3437	20	Jiangnan University	53	2.14%
10	Zheng P (Chongqing Medical University)	10	1487	18	Ohio State University	42	1.69%

TC: total citations

**Figure 4:**
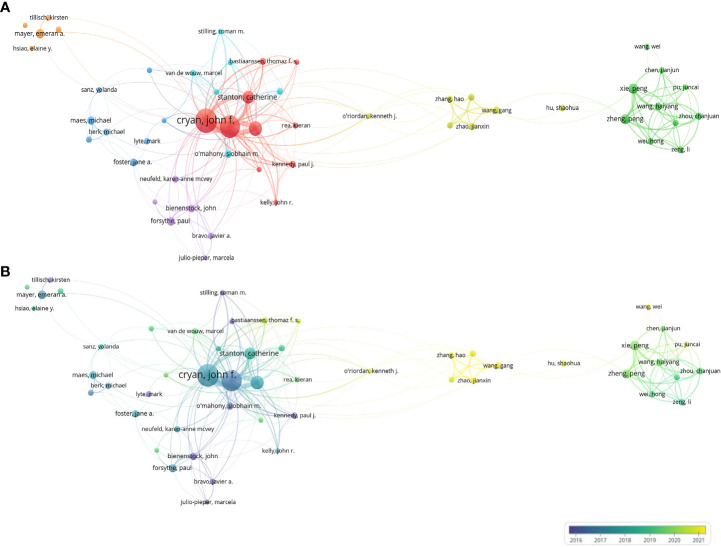
Map of collaboration networks of co-author analysis (**(A)** Network visualization map of authors; **(B)** Overlay visualization map of authors).

**Figure 4** presents the co-author network, which includes 52 authors with a TLS of 1,154, and each author with at least 7 articles. Cryan JF (TLS: 332), Dinan TG (TLS: 299), and Clarke G (TLS: 160) separately shared the strongest collaboration with others. As shown in **Figure 4B**, Wang G (APY: 2020.83; NP: 12; TLS:43), Zhang H (APY: 2020.81; NP: 11; TLS: 37), Chen W (APY: 2020.9; NP: 10; TLS: 40) and Zhao JX (APY: 2020.9; NP: 10; TLS: 40) have published the most publications on brain-gut axis and psychiatric disorders in recent years (yellow color).

**Figure S3** presents the collaborations between institutions, which includes 87 institutions with a TLS of 750, and each institution with at least 10 articles. University College Cork (TLS: 77) had the strongest collaboration with other institutions. **Figure S3A** shows strong cooperation between institutions in the same region, such as Chongqing Medical University and the Third Military Medical University of Chinese in Chongqing, China; the University of Melbourne (Melbourne, Australia) and Deakin University (Victoria, Australia). **Figure S3B** shows that institutions from China have been active in the brain-gut axis and psychiatric disorders research in recent years (yellow color), such as Ningbo University (APY: 2021.36; NP=12), Qingdao University (APY: 2021; NP=10) and Southern Medical University (APY: 2020.67; NP=12). Further, **Figure S4** shows the collaborations between authors and institutions on certain topics. For example, Cryan JF published the most articles with the keywords “gut-brain axis”, “gut-microbiota”, “microbiota”, and “depression” through University College Cork.

### 3.5 Most cited articles

**Table S3** shows the top 10 most cited articles on the brain-gut axis and psychiatric disorders with the citations ranging from 805 to 2,161. Eight of them were published in journals classified as Q1 and with IFs greater than 10. The article entitled “*Mind-altering microorganisms: the impact of the gut microbiota on brain and behaviour*” in Nature Reviews Neuroscience (IF=38.755; Q1) had the most citations (N=2,161) (44).

### 3.6 Keywords

#### 3.6.1 Keywords co-occurrence networks

**Figure 5:**
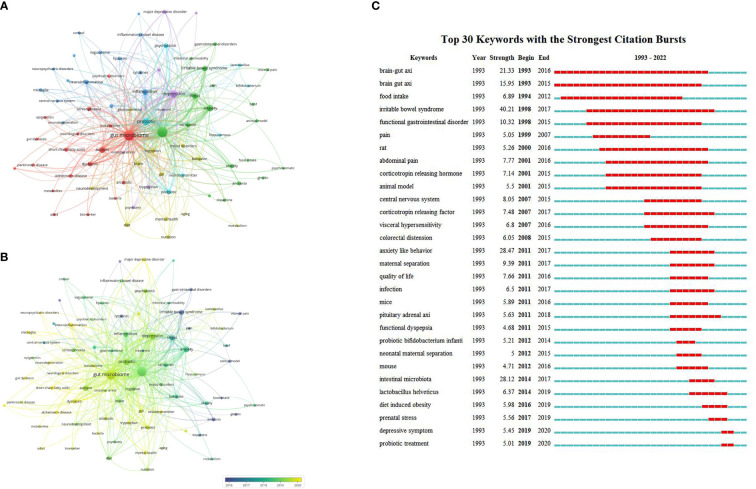
Analysis of the research hotspots on brain-gut axis and psychiatric disorders (**(A)** Network visualization map of keywords co-occurrence; **(B)** Overlay visualization map of keywords; **(C)** Top 30 keywords with the strongest citation bursts from 1993 to 2022).

Of the 3,599 keywords extracted from the 2,298 articles, 78 keywords that occurred more than 15 times were analyzed with 7,984 TLS (**Figure 5A**). “Gut microbiome” (1,013), “brain-gut axis” (880) and depression (353) were the most frequent keywords. The most frequently used keywords were collapsed into six clusters. Cluster 1 (red color) mainly refers to the gut microbiome and neurodegenerative & neurodevelopmental diseases such as “gut microbiome”, “autism”, “ADHD”(Attention deficit hyperactivity disorder), “Alzheimer’s disease”, “neurological disorders” and “Parkinson’s disease”. Cluster 2 (green color) refers to the brain-gut axis and stress-related diseases such as “anxiety”, “stress” and “irritable bowel syndrome”. Cluster 3 (deep blue color) refers to immune-related topics such as “inflammation”, “neuroinflammation” and “immune system”. Cluster 4 (yellow color) refers to brain behavior-related topics such as “cognition”, “behavior”, “brain” and “neurodevelopment”. Cluster 5 (purple color) refers to depression-related topics such as “depression”, “major depressive disorder”, and “bipolar disorder”. Cluster 6 (light blue color) refers to probiotic-related topics such as “probiotic”, “prebiotic” and “psychobiotic”. **Figure 5B** shows that depression (880; APY: 2019.26), anxiety (207; APY: 2018.31) and autism (191; 2019.28) were the most studied psychiatric disorders in the past 5 years.

#### 3.6.2 Keywords with the strongest citation bursts

**Figure 5C** shows the top 30 keywords that had the strongest citation bursts with a minimum duration of 2 years during the period from 1983 to 2022. The keywords “brain-gut axis” (1993-2016) and “irritable bowel syndrome” (1998-2017) had gained the most sustained interest in the earlier period. In contrast, “depressive symptoms” (2019-2020) and “probiotic treatment” (2019-2020) were the main areas addressed in recent years, indicating that these areas are potential future research frontiers in the field of the brain-gut axis and psychiatric disorders.

## 4 Discussion

To the best of our knowledge, this was the first bibliometric study to provide a comprehensive review of the developing trends of research on the brain-gut axis and psychiatric disorders. Overall, 2,298 publications were identified between 1993 and 2022. The number of publications on the brain-gut axis and psychiatric disorders has rapidly grown, especially during the past decade, which is in line with the overall trend of the brain-gut axis (45, 46) and microbiome research (47–49). The annual publication numbers pattern included a stable (before 2013) and rapid growth period (2013 and afterward), indicating that the exploration of the brain-gut axis and psychiatric disorders is an emerging hot research topic (45). This may be related to the release of the second phase of the Integrative Human Microbiome Project (iHMP) funded by the National Institutes of Health in 2013 (50). Some studies have predicted that the brain-gut axis and psychiatric disorders will continue to gain strong attention in the coming decade (45).

With the initiative of the Microbiome Project (50, 51), research outputs on the brain-gut axis and psychiatric disorders have been published in multiple countries, with China being the most productive country, followed by the USA and Ireland. This finding is not completely consistent with the overall research on the brain-gut axis as a field, which showed that the USA contributed the most publications, followed by Ireland and China (46). This may indicate that China has substantially increased research activities related to the brain-gut axis and psychiatric disorders. Adequate research funding is required to achieve large scientific research outputs. In this study, except for China, all others in the top 10 productive countries are in the high-income level. As an upper-middle income level country, China has invested huge financial support in microbiology research with the launch of the Microbiome Initiative (CMI) by the Chinese Academy of Sciences in 2017, including incremental funding for the National Natural Science Foundation of China, the “973” program, the “863” program and the special projects of the Chinese Academy of Sciences (51). Liu et al. reported that China’s research and development expenditure in the field of microbiology is now almost 400 million yuan (approx. USD 57.8 million) per year (51). Publications from Ireland had the most significant impact, which may be due to the fact that the most active authors came from Ireland (46, 49), specifically from the Alimentary Pharmabiotic Centre (APC) of University College Cork, with funds by the Science Foundation Ireland (SFI) and several collaborative companies (46).

Journal analysis can help researchers select appropriate journals for their research outputs. In this study, the journals Nutrients, Brain Behavior and Immunity, and Frontiers in Psychiatry were the most productive journals on the brain-gut axis and mental disorders. Previous bibliometric analyses on gut microbiota and depression (49) or the brain-gut axis (46) showed that Brain Behavior and Immunity was the most productive journal, with an IF increasing from 6.170 in 2018 to 19.227 in 2022; it was ranked 6 out of the 274 journals in *Neurosciences* (46). In addition, the top 10 most active journals published less than 20% of the total number of publications on the brain-gut axis and psychiatric disorders, suggesting a dispersed distribution of publications across journals, probably because brain-gut axis and psychiatric disorders involve multiple research areas including microbiology, clinical neurology, neurosciences, psychiatry, immunology, biochemistry & molecular biology, nutrition & dietetics, pharmacology, and gastroenterology & hepatology, endocrinology & metabolism (49).

The research team at the University of College Cork contributed more than 20% of publications to the area of brain-gut axis and mental disorders, which was consistent with their outputs reported in other studies, such as microbiome and IBS (52), depression (49), and obesity (48). The most influential authors in leading research on the brain-gut axis and psychiatric disorders from University College Cork with strong collaborations with other research fields involved gastroenterology, microbiology, and neurology, all of which provided new perspectives on the intervention and treatment of conditions such as depression, ASD, and obesity (45). In addition, they first proposed and strengthened the research on “psychobiotic” (53), which contributed to the growing publications on the brain-gut axis and psychiatric disorders.

The collaborative networks showed a trend of homophily characterized by close collaboration between institutions within the same region or between authors from the same institution. The time evolution maps can capture the authors or institutions of earlier and emerging studies; for example, Ningbo University started the relevant studies in the past two years. In addition, this study showed a collaborative relationship between authors and institutions on a specific topic, which helps to understand the researchers’ directions (54).

The most cited articles reflect the most valuable and influential findings that can further guide the research direction in one field. The article “*Mind-altering microorganisms: the impact of the gut microbiota on brain and behaviour*” published in Nature Reviews Neuroscience was the highest cited article (44), with citations growing 1.8-fold from 2019 (1,204) to 2022 (2,161). This article proposed that the concept of a brain-gut axis may lead to the development of novel therapeutics for the management of several neurological and psychiatric disorders (44). The second highest cited article found that the lactobacillus strain could regulate emotional behavior and central GABA receptor expression in a mouse model, indicating a specific strain of bacteria can play a therapeutic role in stress-related disorders (55).

The hotspot analysis showed that psychiatric disorders related to the brain-gut axis mainly focused on neurodegenerative diseases (e.g., Alzheimer’s disease, Parkinson’s disease), neurodevelopmental disorders (e.g., ASD, ADHD), and stress-related diseases (e.g., anxiety, depressive and bipolar disorders), of which, depression, anxiety and ASD were the most studied psychiatric disorders. Clinical and preclinical studies have implicated multiple mechanisms in the bidirectional pathways between certain psychiatric disorders and the brain-gut axis including the activation of immune response, the dysfunction of HPA, and altered neurotransmitter or neuromodulators levels (2, 9, 56). Anxiety-like behavior is often parallel to depression-like behaviors (9). Evidence also indicated that the dysbiosis of gut microbiota could increase the vulnerability to develop depression or anxiety, while depressive or anxiety symptoms could be induced by fecal transplantation (9, 21, 56). Further, the link between ASD and gut microbiota through the brain-gut axis has been widely studied. Changes in the composition of the gut microbiota and microbial metabolites may be associated with the development of ASD and its severity, which may be relevant in the diagnosis, prevention, and targeted intervention of ASD (9).

Furthermore, we found that the evolution of topics started from GI-related problems (e.g., IBS, gastrointestinal disorders, anorexia, abdominal pain, and food intake) in the earlier period to neurodegenerative & neurodevelopmental diseases (e.g., Parkinson’s disease, ASD, ADHD, Alzheimer’s disease) in recent years, indicating that gut-to-brain pathways have been of more recent focus. We also found that gut microbiome was the most frequent keyword, further confirming its pivotal role in the bidirectional pathway of the brain-gut axis. Earlier studies found alterations in the gut microbiota composition in patients with psychiatric disorders; however, the cause-effect relationship remains controversial. It is unclear whether dysbiosis of the gut microbiota is the cause or the result of a specific disorder, or a combination of both (9). Furthermore, the specificity of gut microbiota determines its different changes and roles in individual disorders, which increases the uncertainty of clarifying this cause-effect relationship (9).

The cluster of immune-related topics reflects the important role of the immune system on the bidirectional pathway of the brain-gut axis. Previous studies have addressed various mechanisms of the brain-gut axis in psychiatric disorders, involving the synergistic effects of neural, endocrine, and immune systems (9, 57). For example, evidence showed that activation of the vagus nerve by cytokines could stimulate neuronal anti-inflammatory responses, and immune cells could produce various neurotransmitters and other factors that regulate mood and behaviors (58). In addition, immune activation or immunomodulation might act on serotonergic systems and thereby exerting influence on physiology and behaviors (58).

Probiotics have been a very recent hotspot and appear to be an emerging research frontier in the field of brain-gut axis and psychiatric disorders, which can represent novel treatment targets for both psychiatric disorders and GI-related diseases (59, 60). Previous studies have revealed the important relationship between probiotics, psychobiotics and cognitive and behavioral processes (61). With the proposed “psychobiotic” theory, probiotics have been viewed as a key element in affective disorders and the immune system (53, 61). For instance, previous findings indicated that psychobiotics may have anxiolytic and antidepressant effects (62–64), which could involve multiple signal pathways such as the ENS and vagus nerve, the immune signal network of interactions between microbe-associated molecular patterns and immune factors (65). Additionally, *Bifidobacterium breve CCFM1025* could have antidepressant-like effects by reshaping the gut microbiota community, reducing inflammation, and rebalancing the HPA axis hyperfunction (66). Furthermore, the benefits of probiotics were also found in Alzheimer’s disease and acute mania (67). However, the effects of probiotics on ASD were inconclusive; some evidence suggested that early intake of probiotics may have a preventive role on ASD (67, 68). Moreover, the positive effects of probiotics in treating schizophrenia are still controversial and need further investigations in future studies (69, 70).

This was the first study to explore the developing trends in the past decades and potential frontiers of research on the brain-gut axis and psychiatric disorders from a bibliometric perspective. However, there are some limitations in this study. Based on the recommendation of bibliometric analysis guidelines (30) that the use of a single database is preferable, the included publications were limited to WoS, which is the largest biomedical database and the most commonly used database for bibliometric analyses (20, 71). In addition, a comparative analysis of the publication characteristics by psychiatric disorder was not performed because multiple psychiatric disorders were often included in identified publications.

In summary, research on the brain-gut axis and psychiatric disorders has grown rapidly, especially in the last decade. China, the USA, Ireland and other high income countries contributed to the most publications in this area. Depression, anxiety, and autism have been the most frequently studied psychiatric disorders in recent years. Probiotic treatment is a potential new therapy direction for psychiatric disorders in this area.

## Data availability statement

The original contributions presented in the study are included in the article/**Supplementary Material**. Further inquiries can be directed to the corresponding authors.

## Author contributions

Study design: PC, LZ, YF, CN, Y-T. Data collection, analysis and interpretation: PC, Y-FL, TS, ZS, TC, GU. Drafting of the manuscript: PC, Y-TX. Critical revision of the manuscript: CN. Approval of the final version for publication: all co-authors. All authors contributed to the article and approved the submitted version.

## Funding

The study was supported by the National Science and Technology Major Project for investigational new drug (2018ZX09201-014), the Beijing Municipal Science & Technology Commission (No. Z181100001518005), and the University of Macau (MYRG2019-00066-FHS; MYRG2022-00187-FHS).

## Acknowledgments

The authors are grateful to all participants and clinicians involved in this study.

## Conflict of interest

The authors declare that the research was conducted in the absence of any commercial or financial relationships that could be construed as a potential conflict of interest.

## Publisher’s note

All claims expressed in this article are solely those of the authors and do not necessarily represent those of their affiliated organizations, or those of the publisher, the editors and the reviewers. Any product that may be evaluated in this article, or claim that may be made by its manufacturer, is not guaranteed or endorsed by the publisher.
