# Supplementary material for: Brain-gut axis and psychiatric disorders: A perspective from bibliometric and visual analysis
Source: Front Immunol. 2022 Nov 16;13:1047007. doi: 10.3389/fimmu.2022.1047007 (PMC9709456; doi:10.3389/fimmu.2022.1047007)
Supplement: Supplementary file 1 [file DataSheet_1.docx]

**Supplementary Materials**

Figure S1. World map for the distribution of publications on brain-gut axis and psychiatric disorders

Figure S2. Top 20 most productive countries of corresponding authors on Brain-Gut Axis and psychiatric disorders

Figure S3. Map of collaboration networks between institutions (A. Network visualization map of institutions; B. Overlay visualization map of institutions)

Figure S4. Three-Fields Plot of the keywords analysis (author-institution-keyword)

Table S1. Number of annual publications on brain-gut axis and psychiatric disorders (N=2,298)

| **Year** | **NP** | **Percent** | **TC/Y** |
| --- | --- | --- | --- |
| 1993 | 2 | 0.09% | 0.81 |
| 1994 | 4 | 0.17% | 2.66 |
| 1995 | 1 | 0.04% | 0.00 |
| 1996 | 2 | 0.09% | 0.85 |
| 1997 | 1 | 0.04% | 0.08 |
| 1998 | 1 | 0.04% | 1.42 |
| 1999 | 5 | 0.22% | 2.05 |
| 2000 | 2 | 0.09% | 3.02 |
| 2001 | 6 | 0.26% | 3.98 |
| 2002 | 5 | 0.22% | 1.16 |
| 2003 | 1 | 0.04% | 2.05 |
| 2004 | 5 | 0.22% | 6.70 |
| 2005 | 3 | 0.13% | 1.75 |
| 2006 | 0 | 0.00% | 0.00 |
| 2007 | 12 | 0.52% | 3.63 |
| 2008 | 11 | 0.48% | 1.89 |
| 2009 | 6 | 0.26% | 18.15 |
| 2010 | 11 | 0.48% | 8.84 |
| 2011 | 29 | 1.26% | 22.11 |
| 2012 | 26 | 1.13% | 20.97 |
| 2013 | 26 | 1.13% | 19.46 |
| 2014 | 62 | 2.70% | 14.51 |
| 2015 | 70 | 3.05% | 15.64 |
| 2016 | 120 | 5.22% | 13.87 |
| 2017 | 161 | 7.01% | 14.85 |
| 2018 | 189 | 8.22% | 10.82 |
| 2019 | 320 | 13.93% | 10.51 |
| 2020 | 393 | 17.10% | 8.63 |
| 2021 | 517 | 22.50% | 7.04 |
| 2022 | 307 (*616*) | 13.36% | - |
| *2023* | *726* | - | - |
| *2024* | *837* | - | - |
| *2025* | *949* | - | - |
| *2026* | *1,059* | - | - |
| *2027* | *1,166* | - | - |
| *2028* | *1,273* | - | - |
| *2029* | *1,380* | - | - |
| *2030* | *1,487* | - | - |
| *2031* | *1,595* | - | - |
| *2032* | *1,704* | - | - |

Notes*: Italics: Predicted numbers of publications.* TC/Y: Average per Year Total Citations

Table S2. Top 10 most productive countries of corresponding authors on brain-gut axis and psychiatric disorders

| **SCR** | **Country** | **Income level** | **NP** | **Percent** | **TC** | **SCP** | **MCP** | **MCP_Ratio** |
| --- | --- | --- | --- | --- | --- | --- | --- | --- |
| 1 | China | Upper middle | 458 | 19.93% | 8,929 | 356 | 102 | 0.223 |
| 2 | USA | high | 436 | 18.97% | 16,129 | 354 | 82 | 0.188 |
| 3 | Ireland | high | 153 | 6.66% | 21,334 | 120 | 33 | 0.216 |
| 4 | Italy | high | 131 | 5.70% | 3,381 | 104 | 27 | 0.206 |
| 5 | Canada | high | 126 | 5.48% | 10,557 | 81 | 45 | 0.357 |
| 6 | Germany | high | 73 | 3.18% | 1,543 | 53 | 20 | 0.274 |
| 7 | Japan | high | 69 | 3.00% | 1,657 | 54 | 15 | 0.217 |
| 8 | France | high | 68 | 2.96% | 3,355 | 43 | 25 | 0.368 |
| 9 | Spain | high | 64 | 2.79% | 1,162 | 47 | 17 | 0.266 |
| 10 | Australia | high | 58 | 2.52% | 2,379 | 32 | 26 | 0.448 |

Notes: SCR: Standard Competition Ranking; TC: Total Citations; AAC: Average Article Citations; SCP: Single Country Publications; MCP: Multiple Country Publications

Table S3. Top 10 most cited articles on brain-gut axis and psychiatric disorders

| **SCR** | **Author & Year** | **Title** | **Journal (IF-2021)** | **TC** | **TC/Y** |
| --- | --- | --- | --- | --- | --- |
| 1 | CRYAN JF, 2012 | Mind-altering microorganisms: the impact of the gut microbiota on brain and behaviour | Nature Reviews Neuroscience (38.755; Q1) | 2,161 | 196 |
| 2 | BRAVO JA, 2011 | Ingestion of Lactobacillus strain regulates emotional behavior and central GABA receptor expression in a mouse via the vagus nerve | Proceedings of the National Academy of Sciences of The United States of America (12.779; Q1) | 1,862 | 155 |
| 3 | FOSTER JA, 2013 | Gut-brain: how the microbiome influences anxiety and depression | Trends In Neurosciences (16.978; Q1) | 1,158 | 116 |
| 4 | BERCIK P, 2011 | The intestinal microbiota affect central levels of brain-derived neurotropic factor and behavior in mice | Gastroenterology (33.883; Q1) | 971 | 81 |
| 5 | CLARKE G, 2013 | The microbiome-gut-brain axis during early life regulates the hippocampal serotonergic system in a sex-dependent manner | Molecular Psychiatry (13.437; Q1) | 961 | 96 |
| 6 | CRYAN JF, 2019 | The Microbiota-Gut-Brain Axis | Physiological Reviews (46.5; Q1) | 888 | 222 |
| 7 | COLLINS SM, 2012 | The interplay between the intestinal microbiota and the brain | Nature Reviews Microbiology (78.297; Q1) | 882 | 80 |
| 8 | O'MAHONY SM, 2015 | Serotonin, tryptophan metabolism and the brain-gut-microbiome axis | Behavioural Brain Research (3.352; Q2/3) | 849 | 106 |
| 9 | ZHENG P, 2016 | Gut microbiome remodeling induces depressive-like behaviors through a pathway mediated by the host's metabolism | Molecular Psychiatry (13.437; Q1) | 839 | 120 |
| 10 | NEUFELD KM, 2011 | Reduced anxiety-like behavior and central neurochemical change in germ-free mice | Neurogastroenterology And Motility (3.96; Q2/3) | 805 | 67 |


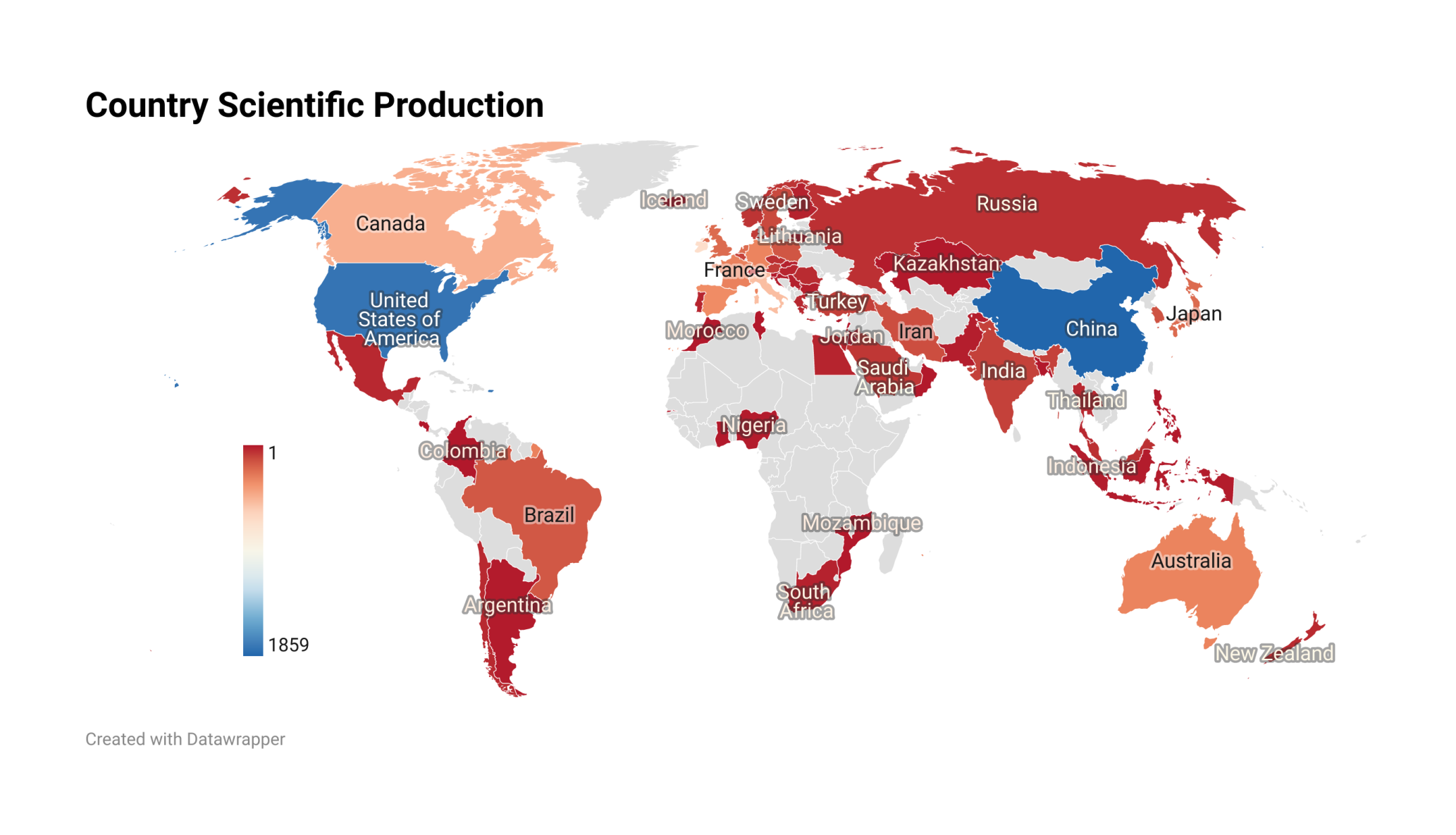


Figure S1. World map for the distribution of publications on brain-gut axis and psychiatric disorders


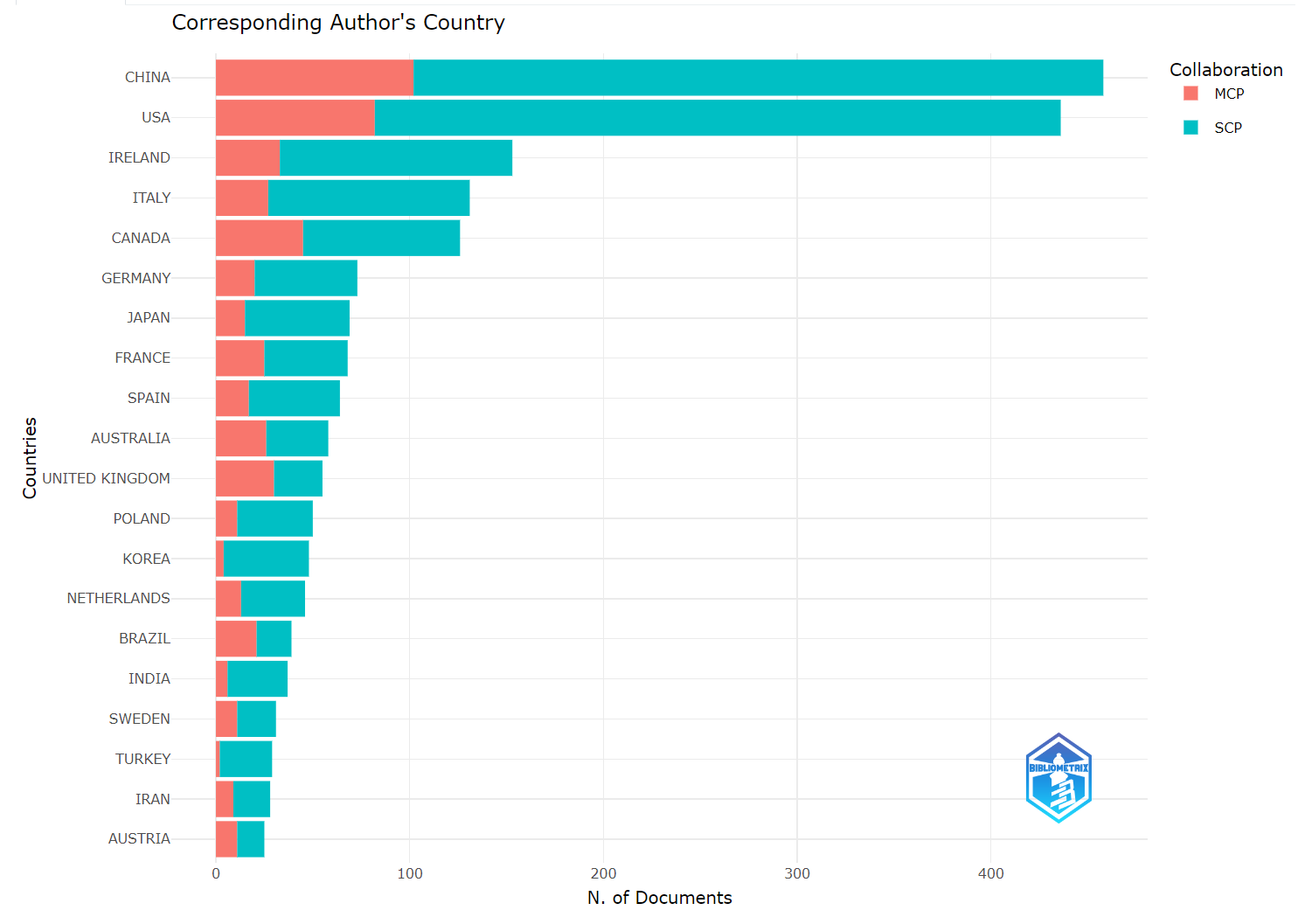


Figure S2. Top 20 most productive countries of corresponding authors on brain-gut axis and psychiatric disorders


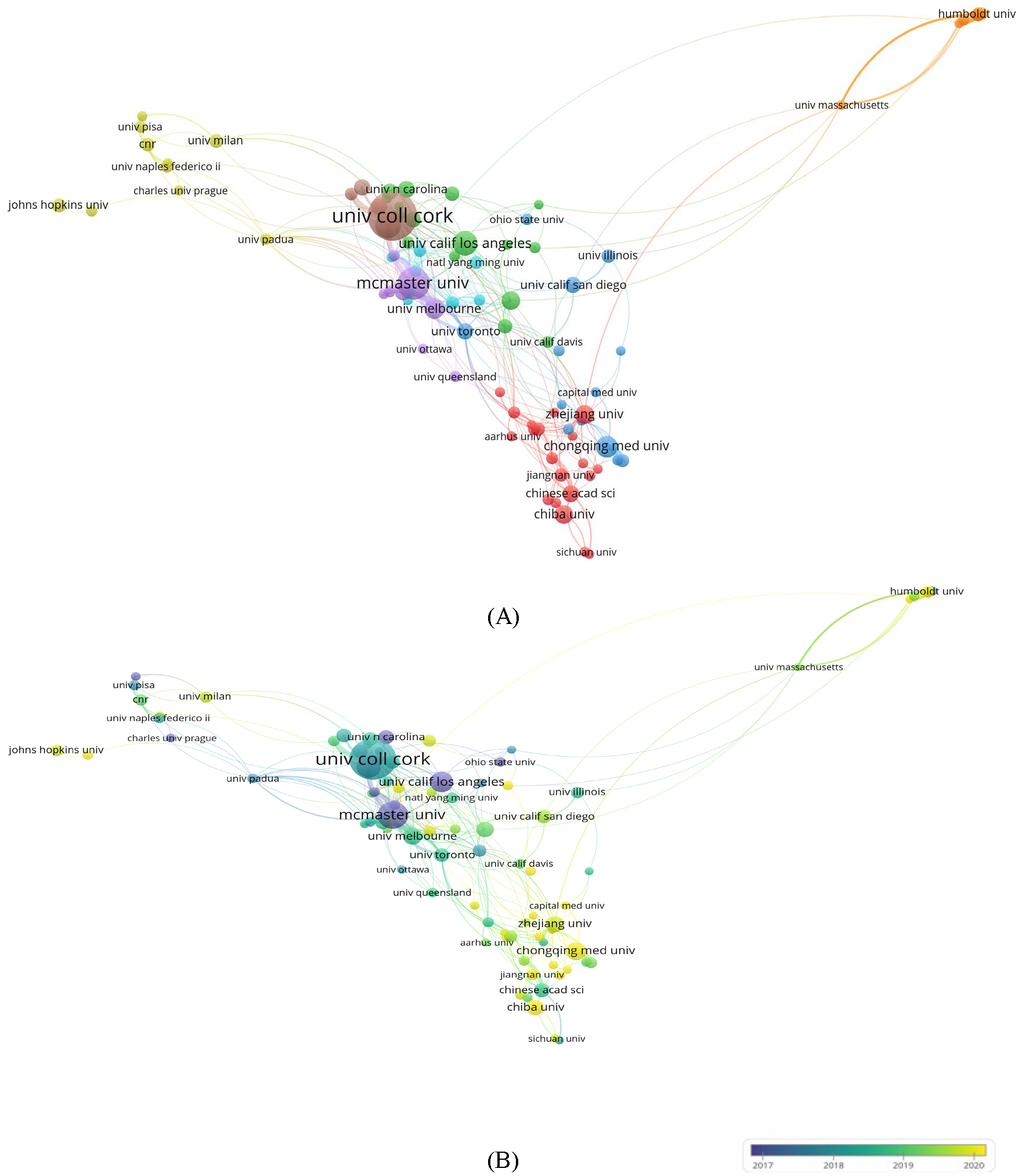


Figure S3. Map of collaboration networks between institutions (A. Network visualization map of institutions; B. Overlay visualization map of institutions)


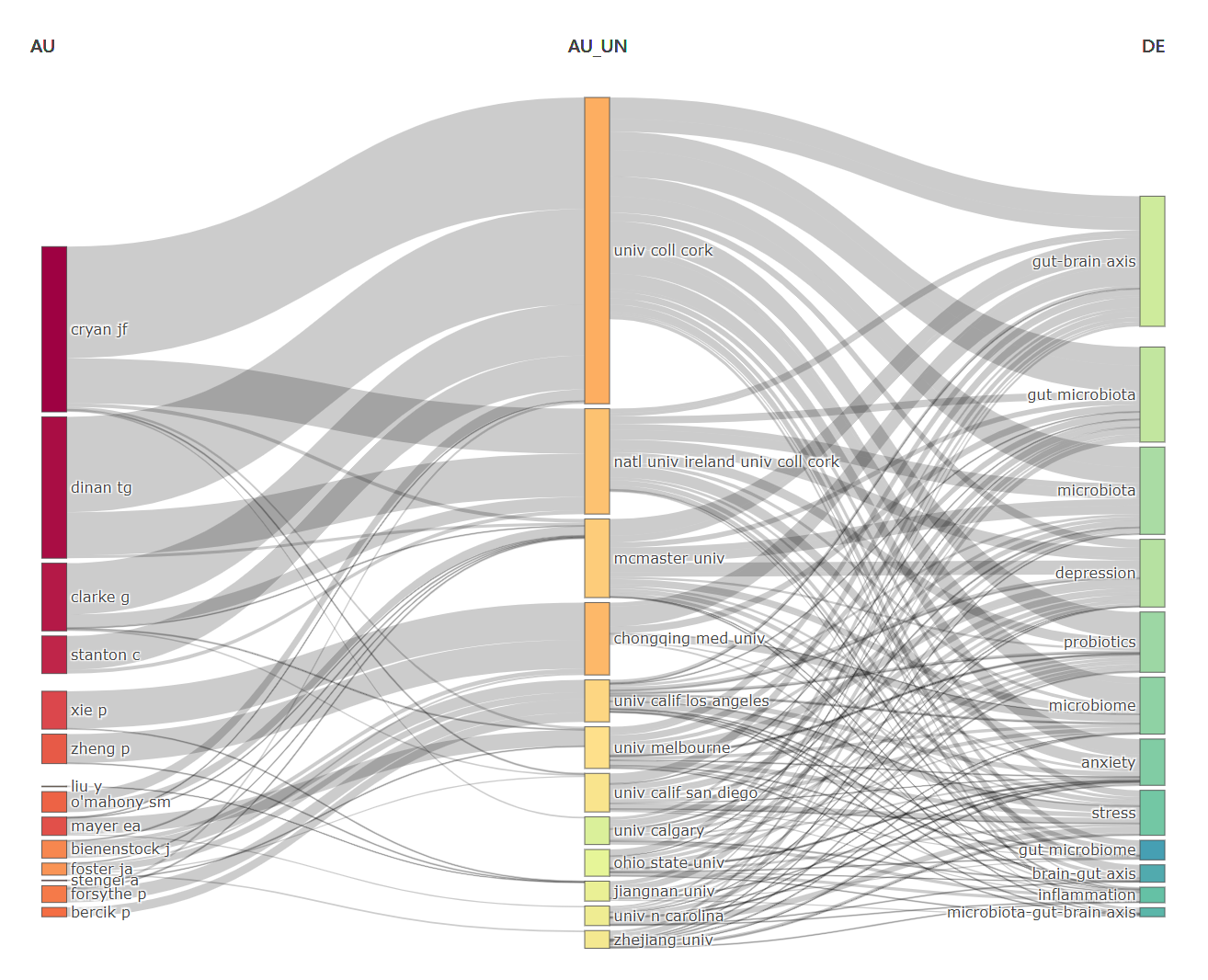


Figure S4. Three-Fields Plot of the keywords analysis (author-institution-keyword)
